# Blood Transcriptional Signatures for Disease Progression in a Rat Model of Osteoarthritis

**DOI:** 10.1155/2017/1746426

**Published:** 2017-07-03

**Authors:** Michał Korostyński, Natalia Małek, Marcin Piechota, Katarzyna Starowicz

**Affiliations:** ^1^Department of Molecular Neuropharmacology, Institute of Pharmacology Polish Academy of Sciences, Smetna 12, 31-343 Krakow, Poland; ^2^Department of Pain Pharmacology, Laboratory of Pain Pathophysiology, Institute of Pharmacology Polish Academy of Sciences, Smetna 12, 31-343 Krakow, Poland

## Abstract

Biomarkers of osteoarthritis (OA) that can accurately diagnose the disease at the earliest stage would significantly support efforts to develop treatments for prevention and early intervention. We have sought to determine the time course of alterations in peripheral blood gene expression profile associated with the development of OA. Blood samples were collected from a tail vein of individual rats with monosodium iodoacetate- (MIA-) induced OA (2, 14, 21, and 28 days after the treatment). We used whole-genome microarrays to reveal OA-related transcriptional alterations of 72 transcripts. Three main groups of coexpressed genes revealed diverse time-dependent profiles of up- and downregulation. Functional links that connect expression of the gradually downregulated genes to the G13 signaling pathway were indicated. The mRNA abundance levels of the identified transcripts were further analyzed in publicly available gene expression dataset obtained from a GARP study cohort of OA patients. We revealed three-gene signature differentially expressed in both rat and human blood (TNK2, KCTD2, and WDR37). The alterations in expression of the selected transcripts in peripheral blood samples of the patients indicate heterogeneity of the OA profiles potentially related to disease progress and severity of clinical symptoms. Our study identifies several potential stage-specific biomarkers of OA progression.

## 1. Introduction

Osteoarthritis (OA) is a common disease associated with damage to the cartilage and resulting in the development of bony spurs and cysts at the margins of the joints. It is characterized by progressive loss of function in the synovial joints. There are a variety of factors that play an important role in the pathogenesis of OA, including biomechanical factors, proinflammatory mediators, and proteases [1–4]. The primary symptoms of OA are joint pain, stiffness, and locomotor restriction. There is no laboratory test that is specific for osteoarthritis. Its diagnosis relies mainly on clinical symptoms, physical, and radiographic findings [5]. The severity of knee OA is classified into four different stages by using the Kellgren and Lawrence system (grades 0–4) [6]. The different stages are distinguished on radiological evidence of synovitis, joint space, and capsular laxity. Identifying patients in the presymptomatic stage would allow the introduction of novel disease-modifying therapies [7, 8].

Blood-based gene expression profiling analysis is useful in discovering RNA biomarkers associated with diverse pathological conditions and disease subgroups [9, 10]. Gene expression studies performed in human blood have indicated networks of coexpressed transcripts with different abundance levels between OA and healthy patients [11, 12]. The results showed relevance to inflammation and cell apoptosis pathways as etiological factors for OA onset and development [11, 13]. The initial results confirm that the blood expression profiles may potentially serve as molecular markers for early detection of OA. However, the clinical heterogeneity and relatively low number of the cases provided limited power to detect differences at the genome-wide scale and identify biomarker candidates as new targets for therapy that will go beyond symptomatic relief to slowing or stopping the progression of OA [14].

The purpose of animal models of OA is to reproduce the pattern and progression of degenerative damage in a controlled fashion. The models provide unique possibility for sequential evaluation of the OA-related transcriptional alterations, including early and intermediate stages of disease [15, 16]. Moreover, the changes in blood gene expression may also indicate physiological processes associated with OA progression. The role of several factors in OA pathogenesis have been suggested based on the increased levels in peripheral blood samples [17, 18]. The molecules considered as promising biomarkers include hyaluronan, collagen epitopes, aggrecan, and matrix metalloproteinase-3 [19]. These changes are accompanied by the sequence of alterations on the molecular level. Therefore, we postulated that transcriptional alterations detected in circulating blood cells might be useful as an additional biomarker of OA.

Here, we report blood gene expression profiles associated with the development of OA in rat MIA model of OA. The dynamics in pattern alterations were monitored in each individual animal during the time course of OA progression [13]. The relevance of the identified transcripts was further examined in data taken from expression profiling in peripheral mononuclear cells obtained from patients diagnosed with OA. The combination of results from animal model and human transcriptome profiling may provide potential new targets for early disease treatment and prevention [14].

## 2. Materials and Methods

### 2.1. Animals

Male Wistar rats (Charles River, Germany) initially weighing between 225 and 250 g were used for all the experiments. Animals were housed 5 per cage under a standard 12/12 h light/dark cycle and had free access to food and water. The procedures on animals were performed following the recommendations of the International Association of Studies on Pain and 3R policy. The study was approved by the Local Bioethics Committee of the Institute of Pharmacology PAS (approval number 938/2012). In the experiments, we compared nontreated (intact) and osteoarthritic animals. Our previous results showed no significant changes in joint hypersensitivity and DWB tests for saline-injected rats (unpublished data). Our study was aimed at the comprehensive comparison between healthy and OA-affected cartilage in respect to the “3R” rules. Rats were randomly allocated to the groups.

### 2.2. MIA-Induced Model of Osteoarthritis

The rats were deeply anesthetized with 5% isoflurane (Forane®, Baxter Healthcare Corporation, USA) in 100% O_2_ (3 L/min) until the flexor withdrawal reflex was abolished. The skin covering the right knee joint was shaved and swabbed with 100% ethanol. A 27-gauge needle was introduced into the joint cavity through the patellar ligament, and 50 *μ*l containing 1 mg of sodium monoiodoacetate (MIA; Sigma-Aldrich, USA) in 0.9% saline was injected intra-articular (i.a.) to induce OA-like lesions. The MIA model was chosen due to its particular usefulness for studying joint pain and multiple similarities in the mechanisms of cartilage degeneration to those observed in human OA cartilage. The dose of the MIA (1 mg i.a. injection) was selected based on previous investigations in rats [15, 16, 20].

### 2.3. Blood Collection and RNA Isolation

Blood samples were collected from the tail vein before (time point: 0) and 2, 14, 21, and 28 days post-MIA injection (time points: 2, 14, 21, and 28). Six replicate samples were analyzed per experimental group. Collected samples were placed in individual tubes and stored at −70°C until RNA isolation. RNA was isolated following the manufacturer's protocol and further purified using the RNeasy Mini Kit (Qiagen Inc.). The total RNA concentration was measured using an ND-1000 Spectrometer (NanoDrop Technologies Inc., Montchanin, DE, USA).

### 2.4. Microarray Analysis

The quality of RNA was determined by using RNA 6000 Nano LabChip Kit and Agilent Bioanalyzer 2100 (Agilent, Palo Alto, CA, USA). Preparation of cRNA was performed according to the protocol provided by Affymetrix (Santa Clara, CA). The total RNA from individual animal was further purified by using RNeasy Mini Kit (Qiagen Inc., Valencia, CA, USA). Total RNA (3 *μ*g) was converted to double-stranded cDNA using SuperScript System (Invitrogen, Carlsbad, CA) and an oligo (dT24) primer containing a T7 RNA polymerase promoter site. Biotin-labeled cRNA was synthesized from cDNA using a labeling kit and purified by using a GeneChip Cleanup Sample Module (Qiagen Inc., Valencia, CA, USA). The yield of the in vitro transcription reaction was determined by product absorbance at 260 nm measured by NanoDrop ND-1000 (NanoDrop Technologies, Inc., Montchanin, DE), and a size of cRNA probes was evaluated by using RNA 6000 Nano LabChip Kit (Agilent, Palo Alto, CA, USA). Fragmented cRNA was used for hybridization to GeneChip® Rat Gene 2.0 ST arrays (Affymetrix). Arrays were washed and stained with streptavidin-phycoerythrin (Merck, Darmstadt, Germany) in Fluidic Station 400 (Affymetrix), according to the standard protocol of the manufacturer. The arrays were scanned by using GeneChip Scanner 3000 (Affymetrix).

### 2.5. Microarray Data Analysis

Microarray data was initially processed using GeneChip Operating Software (Affymetrix) with appropriate quality control. After background subtraction, data was normalized using quantile normalization and then log_2_-transformed. The obtained signal was taken as the measure of mRNA abundance derived from the level of gene expression. Microarray data were standardized using a z-score calculation. Statistical analysis was performed by dChip software using ANOVA filtering and followed by correction for multiple testing (estimation of expected false positives). Hierarchical clustering was performed using the measure of Euclidian distance and average distance linkage methods. Cluster visualization was performed using dChip software [21].

### 2.6. Functional Classification and Cell-Type Specific Gene Expression

Gene annotation tool Enrichr was used to identify overrepresented ontological groups among the gene expression patterns and to group genes into functional classifications. Overrepresented terms (WikiPathways 2016 category) were defined by using *P* < 0.05 threshold obtained by using the Fisher's exact test implemented in Enrichr [22].

### 2.7. Validation in Gene Expression Dataset Obtained from OA Patients

The microarray data set (series accession number GSE48556) was downloaded from the Gene Expression Omnibus (GEO) databases (http://www.ncbi.nlm.nih.gov/geo/) [11]. Illumina HumanHT-12 V3.0 gene expression arrays were used to compare gene expression profiles from peripheral blood mononuclear cells (PBMCs) of OA patients and healthy controls [13]. The 45 Illumina microarray probes corresponding to the list of genes regulated in response to MIA treatment in the animal model were identified. The differences in mRNA abundance levels between the two groups were identified by using *t*-test with the permutation-based estimation of false discovery rate implemented in dChip [21].

## 3. Results

### 3.1. Time Course of MIA-Induced Gene Expression Alterations in the Blood

**Figure 1:**
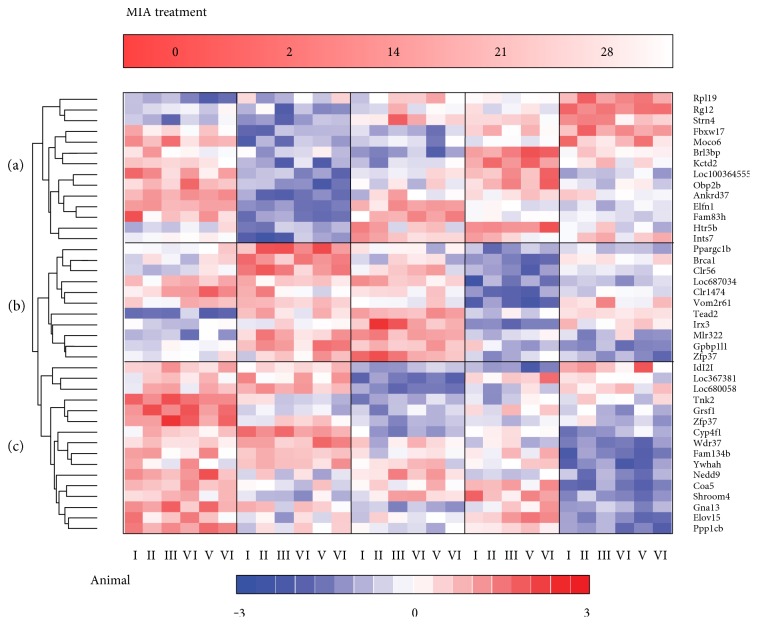
Gene expression patterns in blood associated with OA progression in the rat model of the knee joint arthritis. Hierarchical clustering of MIA-induced transcriptional alterations in whole blood. Microarray results are shown as a heat map and include 41 transcripts with significantly different levels of transcript abundance. Colored rectangles represent transcript abundance 2, 14, 21, and 28 days after the intra-articular injection of the MIA of the gene labeled on the right. The intensity of the color is proportional to the standardized values (between −3 and 3) from each microarray, as indicated on the bar below the heat map image. Hierarchical clustering was performed with the dChip software using Euclidean distance and average linkage method. Major MIA-induced gene transcription patterns are arbitrarily described as A–C. The regulated genes from gene clusters A–C were labeled on the right.

We used whole-genome Affymetrix Rat Gene 2.0 ST microarrays to analyze the time course of gene expression alterations in rat peripheral blood following MIA treatment (1 mg, i.a.). The early, intermediate, and relatively late changes in mRNA abundance were analyzed at four time points (2, 14, 21, and 28 days following MIA injection) and compared to day 0 before MIA treatment (Figure 1). Microarray data analysis using ANOVA filtering (time and treatment as factors, *P* < 0.0001) identified 72 transcripts (expected number of false positives ~4) with the expression level altered during the development of knee joint arthritis (Supplementary File 1 available online at https://doi.org/10.1155/2017/1746426). The further analyses were focused on a list of 41 annotated genes. Interestingly, we found more genes with decreased (20 with a fold change lower than −0.38 at least at one time point of the experiment) than increased (6 with a fold change greater than 0.38 at least at one time point of the experiment) mRNA abundance level after the MIA treatment. The overall number of transcripts regulated throughout the experiment was rather stable 17, 27, 18, and 21 (at the respected time point) (Figure 1).

### 3.2. Gene Signatures Associated with OA Progression

Hierarchical clustering revealed three major gene transcription patterns (arbitrarily described as A–C; Figure 1). Pattern A (14 transcripts) was downregulated 2 days after the MIA treatment and returned close to the basal level after 28 days. The expression of genes with pattern B (11 transcripts) showed an increase in mRNA abundance levels at point 2 days and decreased at later points of the experiment (21–28 days). Pattern C (16 transcripts) consisted of genes displays a decrease of expression during 2–28 days of the OA development. Example genes from the particular patterns include pattern A, *Kctd2*, *Fam83h*, and *Ints7*; pattern B, *Brca1*, *Ppargc1b*, and *Tead2*; and pattern C, *Tnk2*, *Gna13*, and *Ppp1cb*. Three main groups of coexpressed genes revealed diverse time-dependent profiles of up- and downregulation (Figure 1). To identify functional associations between the genes with similar expression profile in blood induced by MIA, we used Enrichr gene list enrichment analysis tool [22]. A list of genes from each gene expression pattern was analyzed by the WikiPathways 2016 categories. Overrepresentation of genes involved in cytoplasmic ribosomal proteins (pattern A, *Rpl19*) and mRNA processing (pattern B, *Brca1* and *Ppargc1b*) was observed. The pattern C was significantly enriched in transcripts connected to G13 signaling pathway (*Ppp1cb*, *Gna13*, and *Tnk2*).

### 3.3. Analysis of the Transcriptional Signatures in Blood of OA Patients

**Figure 2:**
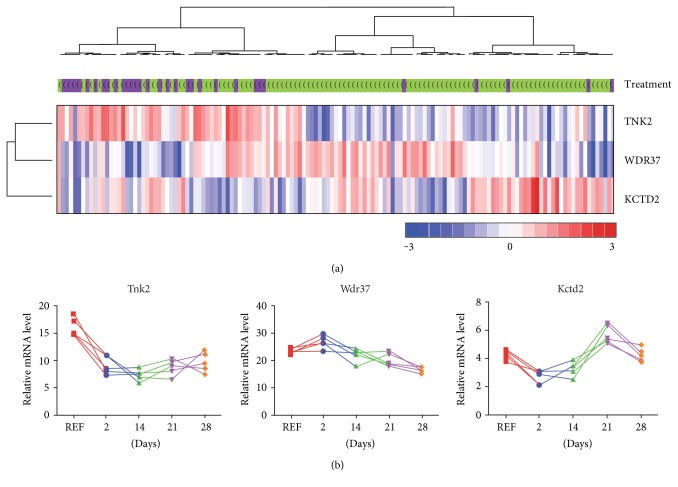
Gene expression profiles of three genes regulated in both animal model and human blood samples. (a) Clustering of the OA patients based on the expression of three genes commonly regulated in the blood of rats and humans. mRNA abundance levels measured by the microarrays were obtained from the GARP study [11]. The dataset consists of blood gene expression profiles of 106 OA patients and 33 controls (OA samples were indicated by green while controls by violet colors). The intensity of the color is proportional to the standardized values (between −3 and 3) from each microarray, as indicated on the bar below the heat map image. Hierarchical clustering was performed with the dChip software using correlation distance metric and centroid linkage method. (b) Time course of gene expression alterations in rat blood of the selected genes. The profiles of individual animals are presented (*n* = 5).

We have analyzed a list of the transcripts (45 microarray probes) regulated by MIA treatment in the microarray dataset obtained from the GARP study [11]. The dataset consists of peripheral blood gene expression profiles of 106 OA patients and 33 controls. We found three differentially expressed genes (TNK2, WDR37, and KCTD2) between the cases and controls (*P* < 0.01). All three transcripts exhibit the same direction of changes in rat model and in patients (Figure 2(b)). The mRNA levels in the PBMCs of OA patient samples reveal high heterogeneity. The separation of OA patients and healthy controls is mostly based on differences in gene expression levels of TNK2. The results provide the additional tool for stratification of the OA patients. The profiles of the identified transcripts indicate potential subgroups of OA patients (Figure 2(a)).

## 4. Discussion

OA is an idiopathic disease with an urgent need for effective therapies and well established molecular biomarkers that can be applied more broadly from the very early to the end stages of knee OA [7]. The chronic progressive process of cartilage loss in a knee joint has complex etiology. Previous studies indicated the connections of disease-associated genes with the inflammatory and apoptotic processes [23]. However, the mechanisms that translate OA-related degenerative and hypertrophic articular changes into the transcriptional responses in the blood are not completely understood. The availability of validated animal disease models gives an opportunity for evaluation of detailed time course of transcriptional changes related to the development of MIA-induced OA [15, 16]. In this study, we confirmed that transcriptional alterations associated with OA can be detected in samples of peripheral blood.

We have monitored transcriptional alterations in the rat model that reproduce time course of OA development. We report a set of 72 transcripts (representing 42 genes) with changes in the mRNA abundance levels during the OA progression. Other studies reveal various numbers of genes with the disease-altered expression in the blood [13, 14, 24]. The transcriptional changes in the peripheral blood produced by OA are subtle comparing to alterations observed in cancer or inflammatory disorders [25]. However, the lower number of genes with altered mRNA abundance levels may provide a more specific molecular signature of cartilage degeneration. Our results indicate that almost all the identified genes were regulated in concert with other genes in the form of three transcriptional modules. The three groups reveal diverse profiles of up- and downregulation of gene expression. The majority of the regulated genes show different patterns of decrease in the expression level during the development of MIA-induced OA. The functional connections of the identified genes may provide novel insight into the possible molecular mechanism of gene regulation associated with the development of OA.

The first group of genes showed rapid downregulation in response to MIA-treatment. The abundance levels of the transcripts went back to control level after 28 days of the experiment. These genes may serve as potential biomarkers of the initial phase of the MIA-induced OA. Surprisingly, in response to MIA injection, we did not find activation in gene expression of factors involved in inflammatory or immunological processes. The genes from this group show heterogeneous biological functions, as for example, *Elfn1* is involved in neuronal transmission [26], *Strn4* is a scaffolding and signaling protein that binds calmodulin [27], and *Ints7* is a subunit of the integrator complex that mediates processing of small RNAs [28]. The second group of coexpressed genes displayed a strong increase in expression 2 days after the treatment, which was later decreased and went back to control level at the last time point of the experiment. Genes from this pattern may indirectly point out molecular mechanisms triggered during the intermediate phase of OA development. The particular genes from this cluster *Ppargc1b* and *Brca1* are associated with the regulation of bone remodeling [29] and binding of steroid receptor hormones [30]. The third identified pattern of transcriptional alterations consisted of gradually downregulated genes. These genes may serve as novel biomarkers of OA progression. In this group, we found a significant enrichment of factors involved in the G13 signaling pathway. G13-mediated signaling was described as the process required for platelet hemostasis and thrombosis [31].

With the aim to validate potential biomarkers of OA progression, we looked for the common molecular changes observed in both, the animal model and OA patients [11, 12]. We reevaluated the available gene expression dataset from the GARP study to search for genes whose expression levels are significantly changed in both humans and rats [11]. We found three genes (TNK2, WDR37, and KCTD2) which are differentially expressed genes between the cases and controls in both the datasets. The number of genes overlapping between the two experiments is relatively low. The differences in the profile of OA-related transcriptional alterations between the whole blood of rats and human PBMCs may also influence the comparison. However, we observed the same directions of alterations in the level of mRNA abundance for all the three genes in a rat model and in humans. We found relatively high individual variability in the expression level of the identified transcripts between the patients.

By using expression profiles of the three identified genes, it might be possible to distinguish potential subgroups of OA patients. WDR37 protein is a member of the WD repeat protein family involved in a variety of cellular processes, including cell cycle progression, signal transduction, apoptosis, and gene regulation [32]. The upregulation of *Wdr37* was observed in a specific subgroup of OA patients. Interestingly, the *Wdr37* knockout mice (Wdr37^tm1a(KOMP)Wtsi^) exhibit skeleton abnormalities, including vertebral fusion and increased bone mineral content (MGI:1920393). The increase in mRNA expression of the potassium channel tetramerization domain containing protein KCTD2 was noticeable in a different group of OA patients. Based on the time-specific profiles of transcriptional alterations revealed in rats, we can suggest that the changes observed in patients may be connected to specific stages of the disease. The third gene TNK2 encoding nonreceptor tyrosine kinase [33], involved in the G13 signaling, showed decreased expression in the majority of OA patients. Further studies are required to indicate the functional meaning of the observed changes in gene expression associated with the development of OA.

Several potential limitations should be acknowledged in the current study. The transcriptional alterations detected in the blood provide indirect indicators of pathophysiological conditions linked to the development of knee OA. The limitations of our strategy also include potential differences in OA etiology between human and animal model. Still, it is necessary to identify biomarkers of OA in preclinical research that can be applied more broadly from the very early stage to the end stage of knee OA and to design better management systems for patients with knee OA. The molecular differences may occur even if the usability of the MIA-induced model of knee OA in rats has been already validated [44]. Our analysis of molecular changes in both rats and humans was based on publicly available gene expression dataset. The conclusions from this part of the results are limited by no access to the clinical description of the patients. Despite these limitations, the obtained results may provide new biomarkers associated with the development of knee OA. Genetic biomarkers have the potential to detect early changes or to prediagnose in OA and classify the OA itself as shown in cancer. Epigenetic mechanisms in cartilage and osteoarthritis include DNA methylation, histone modifications, and microRNAs [34, 35].

## 5. Conclusions

Gene profiling in blood indicated three major patterns of expression associated with the development of OA in a rat model. The present results suggest that alterations in G13 signaling pathway activity in the blood might be associated with the progression of the disease. The expression changes of the three selected transcripts were also found in OA patients and suggest that the molecular stratification of the patients would be possible. The obtained results indicate heterogeneity of gene expression and potential connections with the clinical profiles of the OA patients. The identified genes may provide novel candidates for stage-specific molecular biomarkers of OA progression.

## Supplementary Material

Supplementary File 1: A table listing the results of the ANOVA filtering (time and treatment as factors with the expression level altered during the development of knee joint arthritis. The second sheet contains lists of the genes from the three gene expression clusters a, b and c

## Data Access

Microarray data are available in the NCBI Gene Expression Omnibus (GEO: GSE99021).
